# Meconium Aspiration Syndrome in Animal Models: Inflammatory Process, Apoptosis, and Surfactant Inactivation

**DOI:** 10.3390/ani12233310

**Published:** 2022-11-27

**Authors:** Daniel Mota-Rojas, Dina Villanueva-García, Andrea Mota-Reyes, Agustín Orihuela, Ismael Hernández-Ávalos, Adriana Domínguez-Oliva, Alejandro Casas-Alvarado, Karla Flores-Padilla, Joseline Jacome-Romero, Julio Martínez-Burnes

**Affiliations:** 1 Neurophysiology, Behavior and Animal Welfare Assessment, Universidad Autónoma Metropolitana (UAM), Mexico City 04960, Mexico; 2 Division of Neonatology, National Institute of Health Hospital Infantil de México Federico Gómez, Mexico City 06720, Mexico; 3 School of Medicine and Health Sciences, TecSalud, Instituto Tecnológico y de Estudios Superiores de Monterrey (ITESM), Monterrey 64849, Mexico; 4 Facultad de Ciencias Agropecuarias, Universidad Autónoma del Estado de Morelos, Cuernavaca 62209, Mexico; 5 Clinical Pharmacology and Veterinary Anesthesia, Facultad de Estudios Superiores Cuautitlán, Universidad Nacional Autónoma de México (UNAM), Mexico City 54714, Mexico; 6 Animal Health Group, Facultad de Medicina Veterinaria y Zootecnia, Universidad Autónoma de Tamaulipas, Victoria City 87000, Mexico

**Keywords:** meconium, pulmonary inflammatory response, apoptosis, animal models, surfactant inactivation

## Abstract

**Simple Summary:**

*Meconium aspiration syndrome* is a pathology that causes hypoxia, acidosis, and neonatal mortality. The mechanisms behind this rely on physiopathology and the interaction of meconium with pulmonary alveolar cells. The inflammatory response, inactivation of surfactant, and processes such as apoptosis or necrosis of alveolar macrophages, epithelial and endothelial cells also participate in this syndrome. In this review, the physiopathology of meconium aspiration syndrome will be discussed in veterinary medicine to understand the inflammatory response and the cellular and biochemical changes at the alveolar level that cause the main outcomes of this pathology.

**Abstract:**

Meconium Aspiration Syndrome is a condition that causes respiratory distress in newborns due to occlusion and airway inflammation, and surfactant inactivation by meconium. This condition has been described in animal species such as canids, sheep, cattle, horses, pigs, and marine mammals. In its pathogenesis, the pulmonary epithelium activates a limited inflammatory response initiated by cytokines causing leukocyte chemotaxis, inhibition of phagocytosis, and pathogen destruction. Likewise, cytokines release participates in the apoptosis processes of pneumocytes due to the interaction of angiotensin with cytokines and the caspase pathway. Due to these reactions, the prevalent signs are lung injury, hypoxia, acidosis, and pneumonia with susceptibility to infection. Given the importance of the pathophysiological mechanism of meconium aspiration syndrome, this review aims to discuss the relevance of the syndrome in veterinary medicine. The inflammatory processes caused by meconium aspiration in animal models will be analyzed, and the cellular apoptosis and biochemical processes of pulmonary surfactant inactivation will be discussed.

## 1. Introduction

Meconium aspiration syndrome (MAS) is a condition that causes hypoxemia, acidosis, and respiratory distress in the newborn, increasing neonatal mortality [1,2]. Meconium contains various substances such as mucopolysaccharides, bile acids, cytokines, cholesterol, and cells that, upon entering the airways, interact with the alveolar epithelium, activate the immune system through chemotactic signaling of neutrophils and their extravasation through the alveolar-capillary membrane, additionally stimulating the production of reactive oxygen species (ROS) and apoptosis [3,4,5,6]. This inflammatory process may be accompanied by pulmonary edema, events that consequently cause neonatal hypoxia and acidosis [7,8].

Given the importance of this disease in different species of veterinary interest, various studies have been carried out to describe the pathophysiological mechanisms of MAS [9,10,11]. An example of MAS pathophysiology is the review by Swarman et al. [12], which describes how meconium aspiration provokes airway obstruction and fetal hypoxia. The same authors and coinciding with others mention that meconium microparticles can interact with the alveolar epithelium inducing a local inflammatory response due to the release of cytokines, such as interleukins (IL) IL-1, IL-6, IL-8, IL-10, and tumor necrosis factor (TNF)-α [13,14]. These interleukins and the additional meconium components interact with angiotensin metabolites, causing alveolar cell destruction and inactivation of the pulmonary surfactant [15,16].

The mechanisms described for this pathology have formed the fundamental basis for strategies to address the MAS [17]. Therefore, this article aims to describe and argue the importance of MAS in veterinary medicine. The lung inflammatory process caused by meconium aspiration in animal models will be analyzed, and the cellular and biochemical mechanism of pulmonary surfactant inactivation will be discussed.

## 2. MAS in Veterinary Medicine

MAS is a condition most frequently described in human newborns, with an incidence from 0.4 to 22% [2,18,19,20]. Of these cases, 5 to 28% are associated with infant mortality when adequate obstetric intervention is not received [21,22]. In animals, this pathology has been reported in canids, cattle, sheep, pigs, and marine mammals [22,23,24]. For example, in newborn piglets, 6% of neonatal mortality is associated with this pathology [25,26], while in dolphins, it is 8% [23], 4% in foals [27], and 1–3% in puppies [28].

The frequency in which this pathology may be present during parturition makes it necessary to understand its pathophysiology to assess low vitality or respiratory distress in animals [29]. Even though some clinicians use the degree of meconium staining of the skin to determine severity, it is a poor predictor [30,31]. Therefore, to achieve a definitive diagnosis or early recognition of MAS, complete knowledge is required regarding the pathophysiological mechanisms that trigger the pulmonary and systemic effects of meconium aspiration.

### Animal Experimental Models in MAS

Researchers have tried to reproduce MAS experimentally in laboratory animals to study the nature and progression of pulmonary lesions under controlled conditions. Different animal species have been used as MAS models, including pigs [32,33,34,35,36,37], dogs [28,38], cats [39], guinea pigs [40], rabbits [41,42,43,44], rats [45,46,47], lambs [24,48] and baboons [49]. Pathophysiology and lesions of MAS in humans have been successfully reproduced in experimental animal models and mainly focused on the study of medical interventions and therapies to reduce the impact on newborns’ vitality and mortality [31].

## 3. Meconium Composition

**Figure 1:**
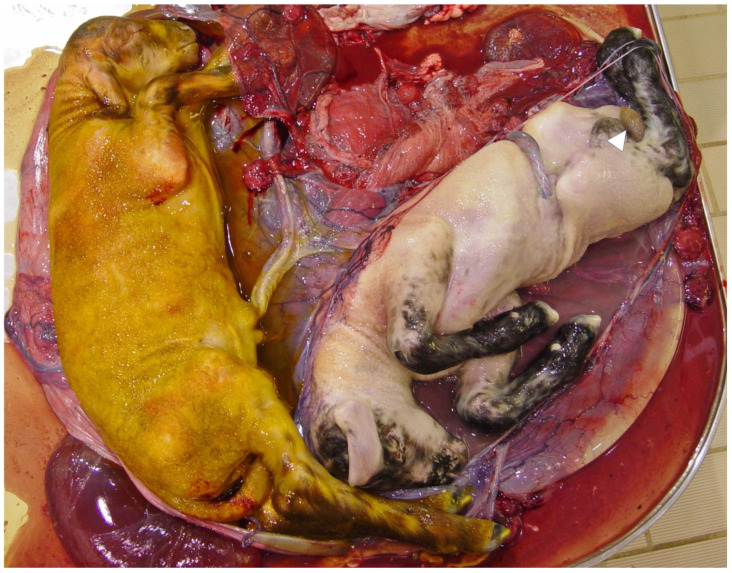
Postmortem inspection of the uterus of a ewe with twin gestation and fetuses that suffered hypoxia. Diffuse severe greenish meconium staining throughout the fetal skin of the left fetus). Large meconium particles expelled from the intestine are on the skin of the second fetus (arrowhead). Courtesy Dr. Carolyn Legge, Atlantic Veterinary College.

**Figure 2:**
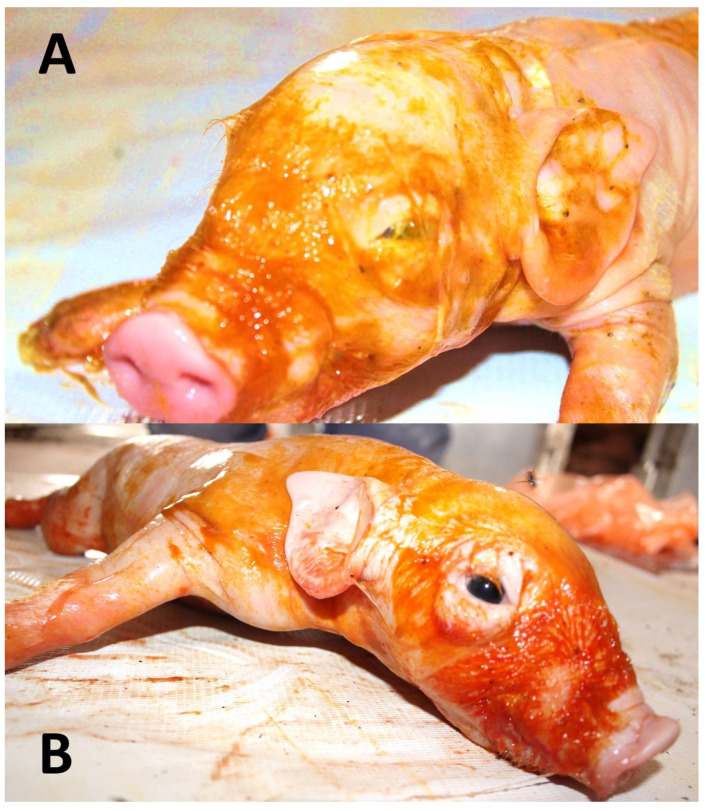
Meconium-stained piglets after dystocia in a primiparous sow. The expulsion interval between neonates was greater than 3 h. Both neonates survived an episode of severe hypoxia. (**A**). The newly born pig was lethargic, adynamic, hypoglycemic, uncoordinated and its vitality score dropped. The newborn was unable to suckle/eat feed as it lost the sucking reflex. It died within the next 24 h. (**B**). Neonate stained with meconium in more than 60% of the body surface. It shows vigor, as it makes attempts to get up despite being weak. After colostrum intake, the piglet survived.

Meconium is a sterile liquid substance or the first excretion accumulated in the fetal intestine during gestation. It has a viscous consistency and green-like color, and it may be expelled from the fetal intestine in response to intrauterine hypoxia [2] (Figure 1 and Figure 2). It is mainly composed of gastrointestinal secretions, biliary salts, pancreatic juice, desquamated epithelial cells, lanugo, amniotic fluid, and blood, in addition to proinflammatory components such as IL-1, IL-6, IL-8, and TNF, as well as proteolytic enzymes such as phospholipase A2, free fatty acids, bilirubin, hemoglobin, and cholesterol. In this sense, Righetti et al. [50], using a proton nuclear magnetic resonance analysis of the components of meconium, found that it is composed of cholesterol and fatty acids, β-glucose, α-glucose, lactate, and β-hydroxybutyrate. These findings allow us to observe the structural and biochemical components of meconium.

Consequently, the components present in the meconium are the main ones responsible for initiating the pulmonary inflammatory response and tissue damage. This event may differ between species, causing the disease mechanisms to be variable, thus leading to a decision of which therapy would be more effective in treating the species.

## 4. MAS and the Pulmonary Inflammatory Process

The systemic inflammatory response induced by meconium inhalation before or during birth is a complex process involving diverse mechanisms of airway damage, such as mechanical airway blockage, epithelial injury, pulmonary hypertension, and surfactant inactivation [14,36]. These lesions involve leukocyte infiltration, activation of alveolar and peritoneal macrophages, myeloperoxidase (MPO) and proinflammatory cytokines release [37,44], cell death by apoptosis, as well as complement and phospholipase A2 activation [5].

The inflammatory process begins as a local lesion in the pulmonary tissue, with an incidence of 13% intraamniotic inflammation and 23% funisitis in the fetus [51]. It can result in a systemic response; however, the importance of the local reaction at the pulmonary level is due to the alterations in the normal physiology of the animal. For example, in the neonatal rat model, airway hyperreactivity leads to neonatal hypoxia, while inflammation of the lung parenchyma can cause atelectasis [2,47].

In the bovine model, a retrospective study analyzing the cause of death in 2-week-old calves showed that 42.5% of the animals had meconium, squamous cells, or keratin in the lung. Histological analysis revealed that the lungs with meconium aspiration developed mild diffuse alveolitis with neutrophils and macrophage infiltration, similar to those reported in humans, by the interaction of cytokines in the pulmonary epithelium [22].

### 4.1. Local Inflammation

Lung damage in patients with MAS is attributed to different mechanisms, such as airway obstruction, chemical pneumonitis, and inflammation due to immune system activation [5,41,52]. Some studies still discuss whether the inflammation is the cause of death of the animal or if it is due to an additional effect of neonatal hypoxia [5,8].

Once the meconium invades the respiratory tract, two innate immune systems are activated: the Toll-like receptor (TLR) and the complement systems [12,41]. Plasma membrane TLRs are highly expressed in lung cells. They identify meconium components and endogenous ligands (e.g., alarmins) released when the tissular damage causes ischemia, as well as events of alveolitis or pneumonitis [8]. According to Anand et al. [53], MAS pathogenesis is associated with the expression of TLR1, TLR4, TLR9, and TLR7 receptors and the initiation of the inflammatory cascade. Upon recognizing foreign or harmful agents, pro-inflammatory substances are activated. For example, TRL3 generates a MyD88-dependent signal that activates the NF-kB and promotes cytokine release [54,55,56].

On the other hand, the activation of the complement system by either of its three pathways (lectin, classical, or alternative) begins after the macrophage infiltration into the pulmonary tissue. The binding of Cq1 activates the classical pathway to antigen-antibody complexes. In contrast, the lectin is activated by mannose-binding lectin or ficolins binding to sugary residues of the antigen to generate a protein complex with the enzymatic activity of the C3 convertase [57]. In vitro studies report that pig meconium causes activation of the alternative pathway [35], which is associated with the activation of some immune mechanisms and the release of IL, as noted by Castellheim et al. [37]. In his experimental study on the lungs of newborn piglets with MAS induction, the levels of a terminal complex sC5b-9 increased significantly compared to the controls (*p* < 0.0005). Likewise, the levels of this complement were significantly correlated with IL-6 (r = 0.64, *p* < 0.005) and IL-8 concentrations (r = 0.32, *p* = 0.03). This finding demonstrates that the activation of the complement system triggers the production of inflammatory mediators such as cytokines, chemokines, eicosanoids derived from arachidonic acid, and ROS due to the interaction between endothelial cells and leukocytes [58]. The terminal sC5b-9 complex releases more C5a, a potent anaphylatoxin that stimulates vacuole degranulation, releasing intracellular and chemotactic mediators [36].

Therefore, the activation of the inflammatory cascade is a process dependent on cell-to-cell communication of the immune system to stimulate the repair of damaged tissue.

### 4.2. Cytokines

**Figure 3:**
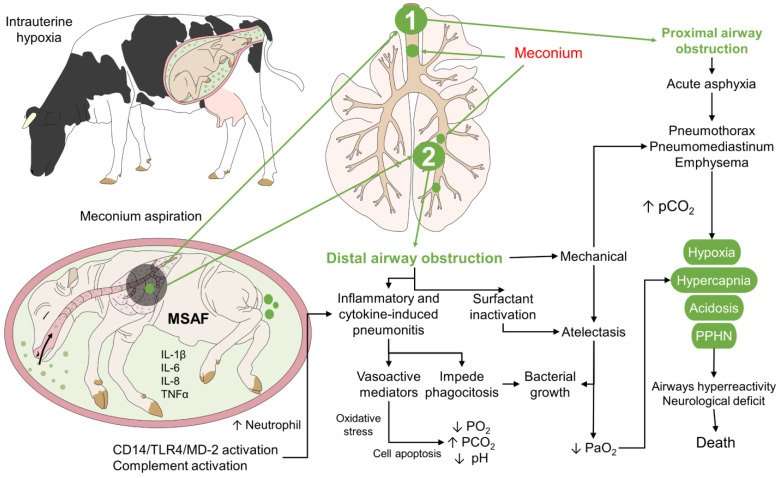
Pathophysiology of meconium aspiration syndrome. In newborns, intrauterine hypoxia is primarily responsible for MSAF and its aspiration. These components generate two primary responses: proximal airway and distal airway obstruction. In the first case, acute asphyxia can lead to pneumothorax, and emphysema, causing hypoxia, hypercapnia, acidosis, and PPHN. Distal airway obstruction induces air trapping and atelectasis. This same effect can be seen after the alveoli’s inflammatory response and surfactant inactivation. In MSAF, proinflammatory cytokines such as IL-1β, IL-6, IL-8, and TNF-α are recognized. The main consequence of prolonged hypoxia is airway hyperreactivity and neurological deficit that can cause neonatal mortality. CD14: a cluster of differentiation 14; IL: interleukin; MD-2: protein MD-2; MSAF: meconium-stained amniotic fluid; PaO_2_: partial pressure of oxygen; PCO_2_: partial pressure of carbon dioxide; PPHN: persistent pulmonary hypertension in the neonate; TLR4: Toll-like receptor 4; TNF-α: tumor necrosis factor-alpha.

Meconium contains different cytokines, such as IL-1, IL-6, IL-8, and TNF-α. However, some authors mention that meconium-stained amniotic fluid contains higher concentrations of these substances, therefore, contained in the amniotic fluid [6,59,60]. These findings support the hypothesis that MAS induces the production of cytokines and the chemotaxis of polymorphonuclear cells and macrophages (Figure 3). In this sense, Lindenskov et al. [13] conducted a study on hypoxic piglets after the pulmonary instillation of meconium and receiving 1.4 mL/kg of 30% albumin. They observed that the levels of IL-8 correlated with the deterioration of pulmonary function measured as the oxygenation index (r = 0.71, *p* < 0.0001), pulmonary compliance (r = 0.66, *p* < 0.0001), and ventilation index (r = 0.71, *p* < 0.0001). These results demonstrate that cytokine induction is closely related to the degree of cell and lung injury.

Neutrophils or macrophages use cytokines as chemical signals to induce cell growth, differentiation, chemotaxis, and cytotoxicity. They respond to ligands with TRLs and chemokines (e.g., IL-8) [14,44,52]. Thus, cytokine signaling indirectly initiates the inflammatory cascade and indicates the type or degree of cell injury.

In this regard, the instillation of meconium in rabbit pups induced a 52% greater number of apoptotic cells than in milk or saline solution (*p* < 0.05). Likewise, the IL-6 and TNF-α were 1.01 ± 0.32 pg/mL and 0.60 ± 0.34 pg/mL higher in the meconium animals [44]. The above reaffirms that cytokine levels could be considered early indicators to recognize MAS or fetal hypoxia. Since some authors have experimentally proven that the presence of albumin with meconium instillation reduces IL-8 expression and inhibits chemotaxis of polymorphonuclear cells, this could be a future treatment for MAS [13].

In an experimental model of MAS in newborn piglets made by Haakonsen Lin-denskov et al. [14], meconium activated the complement system in its C5a’ fraction, activating an inflammasome related to the caspase pathway that, in turn, induced a cellular response through the secretion of cytokines such as TNF-α, IL-1β, and IL-6, released by endothelial cells, T lymphocytes, local alveolar, and peritoneal macrophages. These cells favored phagocytosis and sent activation signals to lymphocytes and monocytes to initiate the local inflammation in the pulmonary parenchyma [61,62,63].

The presence of cytokines in MAS is an essential mechanism in the progression from mild lung disease to severe pneumonia and the development of respiratory distress, cell apoptosis, and severe pulmonary dysfunction.

### 4.3. Phagocytosis

At the pulmonary level, this mechanism begins with the secretion of cytokines that allow the attraction of other inflammatory cells, such as neutrophils and macrophages, to eliminate pathogens at a lesion site [64]. Craig et al. [65] suggest that meconium could inhibit the phagocytic capacity of alveolar macrophages. This finding was also reported in a trial conducted on rat alveolar macrophages NR8383 exposed to sterile human and equine meconium for a short time. In the latter, meconium induced a decrease in phagocytosis of macrophages to fluorescent latex beads but also reduced the presence of the respiratory burst in response to phorbol myristate acetate. These results suggest that contact with meconium alters the alveolar leukocytes’ function and reduces their local defense function [66].

From an immunological point of view, the release of TNF-α by alveolar and peritoneal macrophages can lead to the production of ROS species such as superoxide anion, hydroxyl radicals (OH), as well as non-free radicals such as hydrogen peroxide (H_2_O_2_) and hypochlorous acid (HOCl) that aim to make the phagocytosed meconium by these cells to undergo oxidative digestion that, in combination with the enzymatic digestion of lysosomes, will cause the foreign antigens of the meconium to degrade [37,67]. The aforementioned is of great importance since the oxidative digestion of the meconium components comes from the granulocytes’ respiratory burst. Therefore, macrophages’ normal function is to work as a protective barrier for the organism. However, some studies have shown that meconium at low doses (0.2 mg/mL) tends to inhibit neutrophils and their activity, but at higher doses (1 and 2 mg/mL), it progressively stimulates the production of oxygen radicals in these cells [68]. In such a way, meconium could exert a regulatory function of the immune activity depending on its exposure time and the amount it was exposed to [69].

### 4.4. Apoptosis of Alveolar Cells

**Figure 4:**
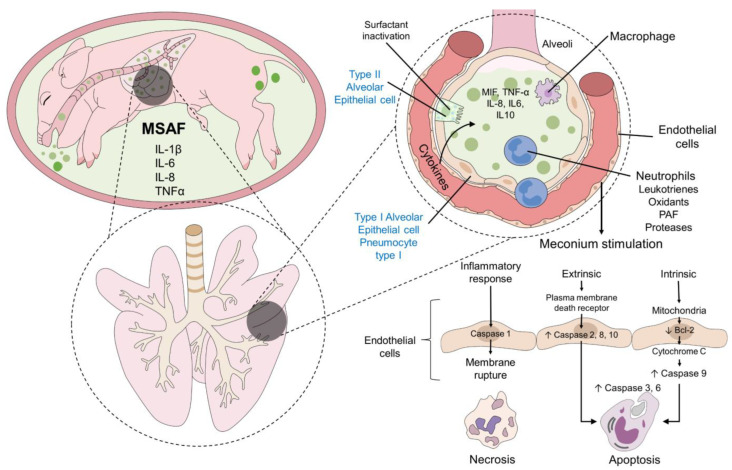
Apoptosis process after meconium aspiration. Derived from the inflammatory process in the lungs and, specifically, in the alveoli, the presence of cytokines, neutrophils, and macrophages evoke the endothelial cells of the alveoli to induce meconium stimulation which can result in three different pathways. The inflammatory response is associated with necrosis of the cells by the intervention of caspase 1. The extrinsic pathway involves the plasma membrane death receptor and the presence of caspase 2, 8, and 10, while caspase 9, together with cytochrome C and Bcl-2, participate in the intrinsic pathway to cause apoptosis of the endothelial cells. Bcl2: b cell lymphoma-2; IL: interleukin; MIF: macrophage migration inhibitory factor; TNF-α: tumor necrosis factor-alpha.

The damage induced by meconium aspiration was corroborated in experimental studies in 7 days-old Fisher rats intratracheally instilled with homologous meconium. Meconium-induced exudative alveolitis, deciliation of pseudostratified epithelial cells, recruitment of neutrophils and pulmonary alveolar macrophages to the bronchoalveolar space, intravascular sequestration of neutrophils, platelet aggregation, interstitial edema, escape of red cells and fibrin into the alveolar space [46,47]. Those findings corroborate that MAS may induce cellular damage and even apoptosis of pulmonary parenchyma cells (Figure 4) [70,71].

In the piglet model inoculated with human meconium, high concentrations of the enzyme phospholipase A2 have been identified, considered a biomarker of cellular injury and trigger of local inflammation [72,73]. Therefore, it is clear that meconium aspiration induces apoptosis of alveolar cells, which is suggested to be the starting point for hypoxia, atelectasis, and hemodynamic changes at the pulmonary level [2].

Although this cellular damage is part of the physiopathology of MAS, the precise mechanism of apoptosis induction is still unclear; a hypothesis suggests that the release of proinflammatory cytokines induces the angiotensin II receptors’ expression, which is associated with cellular apoptosis [52]. In this context, Zagariya et al. [74] corroborated that after meconium aspiration in rabbit lungs and under an in situ end labeling (ISEL-DNA) assay, 70% of apoptotic cells were of the alveolar epithelium. Additionally, 20% of cells were in an apoptosis state with a significant increase of angiotensinogen ARNm, and caspase- 3 expression. Thus, there could be a link between angiotensin levels and apoptosis induction by cytokine activation. It has been reported that levels less than 5 pg/mL of IL-10 can relate to an apoptosis media of 0.26% of neutrophils in newborns [75]. Thus, the above reaffirms the theory that cytokines may induce cellular death by interacting with angiotensinogen.

The explanation for this theory is that cytokines, such as TNF-α, induce the angiotensinogen gene expression leading to the conversion of angiotensin I to angiotensin II through the angiotensin-converting enzyme (ACE), which later binds to the receptors AT-1, linked to caspase pathways activation [15,16,76]. Under this reasoning, it has been postulated that the inhibition or blockade of ACE could inhibit cell apoptosis, as supported by a study by Zagariya et al. [77]. The authors performed the instillation of 10% sterile meconium in 2-week-old rabbit pups and received a dose of captopril at 500 mg/L in the drinking water prior to the instillation of meconium. They observed that captopril significantly reduced the neutrophils and macrophages expressing IL- 13 and IL- 8, IL-4, and TNF-α levels after the meconium inoculation. This could be explained by inhibiting the angiotensin I transformation, which could hinder the pulmonary lesion. Additionally, these authors maintain that captopril reduces leukocyte recruitment capacity due to pulmonary hyperreactivity, which has been refuted in studies using a mouse model [78].

Hence, apoptotic induction of alveolar cells is a crucial mechanism of the pulmonary lesion in MAS physiopathology related to cytokine release and its interaction with angiotensin II. Understanding this interaction could lead to a way to treat and inhibit lung injury and even promote the repair of the pulmonary parenchyma.

## 5. Role of the Surfactant

The lung parenchyma comprises various cells that allow oxygen uptake from breathing and the subsequent elimination of waste in the form of CO_2_ and water vapor. Therefore, it is essential to note that the blood-air barrier is responsible for this process through pneumocytes type 1, 2, and 3. The first two pneumocytes are involved in the barrier formation and can allow the trespass of oxygen from the alveolar space into the bloodstream [3].

### 5.1. Biochemistry and Surfactant Function

The pulmonary surfactant is produced by the pneumocytes type 2 at the alveolar level and by Club cells, formerly known as Clara cells, in bronchioles [79]. This substance is a phospholipid complex (80–85%), neutral lipids (2–5%), and specific proteins (10%). It is worth mentioning that phosphatidylcholine is the most abundant phospholipid of the tensioactive agents representing up to 70% of it. It presents two forms, the union of two palmitic acids to the glycerol-phosphorylcholine or dipalmitoyl-phosphatidylcholine (DPCC). In addition to phosphatidylglycerol (12%), phosphatidylethanolamine (5%), phosphatidylinositol (4%), phosphatidylserine (2%), and sphingomyelin (1.5%) [80,81]. Surfactant is stored in lamellar bodies and secreted by exocytosis to the alveolar lumen during the respiratory dynamic, where it exerts its biological function in the alveolar epithelium [82].

The lung surfactant poses two specific biological functions. The first is reducing the surface tension in the air-liquid interface preventing the alveolar collapse during the gaseous interchange [83]. That is, it stabilizes the interface of water and the alveolus because it prevents the surface tension from approaching zero at the end of expiration, seeking to prevent alveolar collapse [84,85]. In this regard, Schenck and Fiegel [86] characterized the tensiometric behavior of a lung surfactant extract of a calf on the air-liquid interface in viscoelastic gels as similar to the mucus of the pulmonary parenchyma inflammation. The authors observed that the viscoelastic gel property inhibited 0.24 ± 0.42% because of the capacity of lung surfactant to reduce the surface tension.

The second function of lung surfactant is immunity due to the presence of hydrophobic surfactant proteins (SPs), in which surfactant-A (SP-A) and protein surfactant-D (SP-D) are considered calcium-dependent lectins, participating in diverse innate immunity mechanisms in the lung, and in the homeostasis of alveolar surfactant in mammals [85,87]. Han and Mallampalli [82] describe that mentioned proteins allow the elimination of pathogens such as viruses or bacteria. This is due to the presence of domains of C-terminal lectins that bind to pathogen microorganisms’ oligosaccharides. It is described that these collectins facilitate their phagocytosis by monocytes and macrophages as part of the function of the innate immune system [88,89,90].

The presence of collectins in pulmonary surfactant has been the subject of study in experimental models in mice deficient in SP-A, intratracheally inoculated with pathogens such as *Pneumocystis carinii* and group B *Streptococcus*, where the absence of these proteins was associated with an inefficient clearance of these agents [91,92]. Interestingly, other findings were observed in an experiment using mice deficient in SP-A, SP-D, and macrophage TLR receptors, which were associated with a lower presence of connexin-43, IL-1, IL-6, and receptor of oxytocin that caused a 12 h increase in labor time [93]. This means that pulmonary collectins acting via TLR2 serve a modulatory role in the timing of labor, which could play a role in modulating other physiological responses, such as labor.

From all the above, it is evident that pulmonary surfactant has a dual function of maintaining oxygen exchange by reducing surface tension and establishing defense mechanisms against pathogenic microorganisms.

### 5.2. Surfactant and Lung Maturation

Flageole et al. [94] used 17 fetuses from 9 pregnant ewes at term. Fetuses underwent tracheal tamponade at 93 days by tracheal ligation and were unplugged at 110 days. Morphometric analysis revealed that the tamponade induced pulmonary hyperplasia, in addition to a decrease in the number of type II pneumocytes compared to the control group (tamponade = 4.7 ± 0.1 versus control = 55.9 ± 4, *p* = 0.0003). Although the ovine model studies a complete obstruction, the authors denote the need to study the effect of partial obstruction. Therefore, considering the obstructive properties of aspirated meconium, it would be interesting to evaluate the possible intervention in lung maturation in MAS.

#### 5.2.1. Steroid-Associated Regulatory Mechanisms of Lung Maturation

Mammals at the end of the pregnancy experience an increase in the concentration of fetal adrenocorticosteroids, stimulating the Prostaglandin F2α release, and a decrease in the concentration of progesterone by converting it to estradiol, thus promoting luteolysis, and finally, parturition, in addition to lung maturation and surfactant production [95]. A study by Jobe et al. [96] compared the effect of betamethasone acetate (Beta-Ac) and betamethasone phosphate (Beta-PO4) together with Beta-Ac (a combination clinically used in human fetuses) on fetal lung maturation in merino ewes 48 h before premature delivery at different doses, in which it was observed that fetal lung maturation improved even with a single dose of Beta-Ac. In the same ewes, the treatment influenced surface-active proteins, such as SP-A, SP-B, and SP-C, by significantly increasing their synthesis (*p* < 0.05). Results similar to those obtained by Ballard et al. [97] through the application of betamethasone in sheep in a maximum of 4 doses in 104 days of gestation with parturition at 125 days, showing an increase of 80% of body weight from the second to fourth dose in comparison with their control group. Additionally, there was an ~11% increase in SP-A and ~3% SP-B tissue concentrations.

Similarly, it has been recorded that the fetal lung expresses various estrogen receptors (ER), which participate in its maturation by modulating alveologenesis through ERa and ERb, as well as the expression of vascular endothelial growth factor and SP-B and SP-C in conjunction with progesterone [98]. Likewise, Pepe et al. [99] mention that in baboons, the expression of these proteins increases from day 120–140 of gestation, the same period in which lung maturation depends on estrogen and its control by the placental-fetal pituitary-adrenocortical axis.

#### 5.2.2. Biochemical Signaling of Maturation

At the end of gestation, fetuses undergo various changes, such as lung maturation and surfactant synthesis, as adaptive mechanisms to develop and survive in an extrauterine environment [95], in which various biochemical processes mediated by glucocorticoids are involved, sex steroids, insulin, prolactin, catecholamines, fibroblast-pneumocyte factor, and epidermal growth factor [100]. Lung development proceeds in five phases, (1) embryonic, (2) pseudo-glandular, (3) canalicular, (4) saccular, and (5) alveolar. Particularly in dogs, the canalicular phase is observed between days 48 and 57, which is characterized by the development of type I and II pneumocytes, followed by the saccular phase from day 60 until the onset of labor, where it is believed that, like humans, synthesis of pulmonary surfactant takes place [101].

### 5.3. Biochemical Signaling of Surfactant Inactivation

The hypothesis that supports the leading cause of mortality from MAS in the newborn is related to the inflammatory process since the presence of meconium causes a severe local immune response [14]. However, some authors consider an additional mechanism: the inactivation of the pulmonary surfactant. This event could be an essential factor that intervenes in this syndrome’s pathological process, as Sun et al. [42] studied. Various amounts of meconium, the water-methanol soluble fraction, or the chloroform soluble fraction were added to standard suspensions of porcine surfactant (Curosurf^®^). In a pulsating-bubble or Wilhelmy-balance system, meconium and its subfractions inhibited surfactant activity, but the chloroform soluble fraction had the highest specific inhibitory activity. Additionally, using an animal model with rabbits that received sterile human meconium observed that the compliance of the lung-thorax was reduced between 27–38%, and histological analysis showed interalveolar accumulation of fine meconium particles but no plugging in larger airways. These findings demonstrated that the inactivation of the surfactant participates significantly in the mechanisms of disease production, remembering that it tends to maintain the elasticity capacity of the parenchyma, but if it is absent, it could be affected.

**Figure 5:**
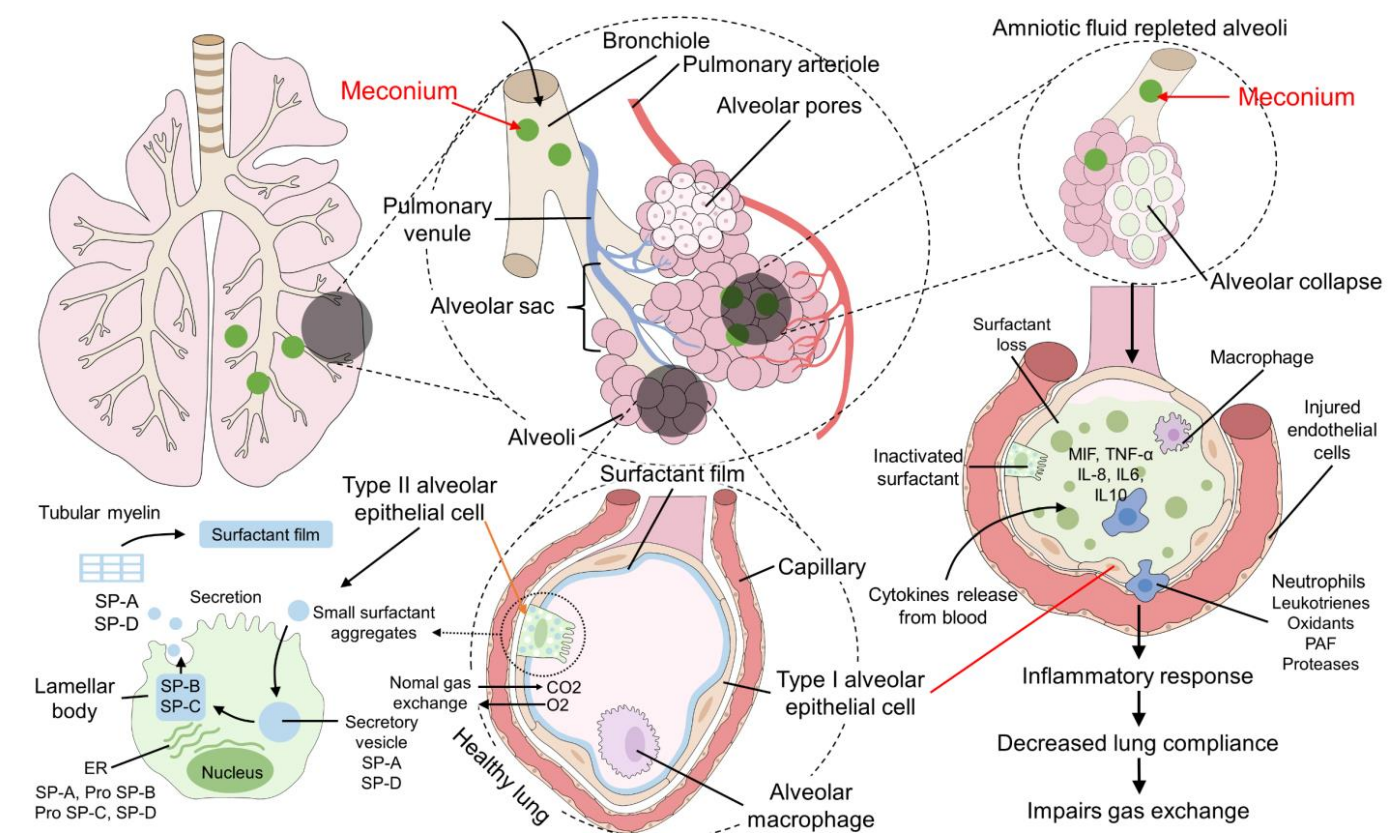
Surfactant inactivation in Meconium Aspiration Syndrome. In a healthy lung, the surfactant film is produced by the type II alveolar epithelial cells (pneumocyte type II) in their lamellar bodies, and this film prevents alveolar collapse. However, when meconium-stained amniotic fluid covers the alveoli, type II alveolar cells are not able to produce surfactant, and the presence of proinflammatory cytokines (e.g., MIF, TNF, IL) promotes the inflammatory response in the lung, as well as a decreased lung compliance, atelectasis, and impairment of the gas exchange, resulting in neonatal hypoxia and acidosis. IL: interleukin; MIF: macrophage inhibitor factor; SP: surfactant protein; TNF: tumor necrosis factor.

Different authors recognize that surfactant deficiency in humans and animals can lead to the onset of respiratory distress or severe respiratory disease due to the loss of the ability to lower surface tension and protect against pathogens [80,102]. Given this evidence, it is necessary to mention how the inactivation of the surfactant by meconium occurs, for which two mechanisms are proposed. The first sustains that meconium components can directly alter the structure of the surfactant, according to Pallem et al. [103], while the second, cited by Kopincova and Calkovska [3], states that the presence of meconium alters the structure of DPCC, which undergoes fragmentation of its bilayer and alters the function of liposomes. Similarly, Casals and Cañadas [104] mention that cholesterol and meconium bile acids together inactivate pulmonary surfactant since bile acid micelles are capable of solubilizing cholesterol, facilitating its transfer to the surfactant complexes, such that their membranes become fluidized, altering their structure and function in interfacial absorption [105]. However, it is also anticipated that bile acids can penetrate and disintegrate lamellar structures and the surfactant monolayer, further affecting its function (Figure 5) [106].

Lugones et al. [107] conducted an in vitro study on the exposure of a natural surfactant to serum proteins, meconium, and cholesterol. They observed at the captive bubble surfactometer test under conditions mimicking respiratory dynamics that surfactant is strongly inhibited by serum exposure, but prior exposure to hyaluronic acid protected them from inhibition. This confirms that components such as serum, cholesterol, and triglycerides can directly inactivate the surfactant and induce changes in its dynamics [108]. For this reason, and supported by animal models in lambs and guinea pigs, it has been suggested that surfactant replacement therapy should be accompanied by a drug that allows its mechanism of action to be carried out successfully [109,110].

Likewise, there is a theory that some compounds derived from the inflammatory process, such as Reactive Oxygen Species (ROS) and soluble Phospholipase A2 (sPLA2), indirectly influence the inactivation of pulmonary surfactants. The latter directly inactivate the surfactant by hydrolyzing its phospholipids, generating proinflammatory eicosanoids and lysophospholipids that induce pneumocyte damage and a secondary inflammatory response culminating in the production of bioactive cytokines and ROS by pneumocytes, macrophages, and neutrophils. Those, in turn, modify the phospholipids and surfactant substances in addition to the destruction of pulmonary surfactant complexes, an effect intensified by the simultaneous production of C-reactive protein, which binds to the membranes of the surfactant, modifying its biophysical characteristics, increasing fluidity, as is the case with cholesterol [111].

Scharama et al. [112] evaluated in vitro the activity of sPLA2 derived from human meconium on the hydrolysis and inactivation of the DPCC of the porcine natural surfactant Curosurf^®^ through the concentrations of lysophosphatidylcholine. After incubation of meconium with the surfactant, the latter hydrolyzed 0.58% of DPCC, but when the temperature increased, this percentage increased to 6.22%, suggesting that the reaction remains stable in the heat. In addition, the surface tension was measured using a pulsating bubble surfactometer, showing an increase when an exclusive PLA2 eluant was applied compared to the eluant composed of other phospholipases, going from 1.7 ± 1.6 mN/m to 19.0 ± 3.58 mN/m (*p* = 0.0001) and 5.9 ± 3.9 mN/m, *p* = 0.07, respectively. In addition, Rodríguez-Capote et al. [113] evaluated the effect of ROS on pulmonary surfactant components by the oxidizing action of hypochlorous acid or Fenton reaction on bovine lipid extract surfactant (BLES) and a synthetic PL and SP-B and SP-C surfactant. A 20% decrease in phosphatidylcholine (PC) was observed after 24 h of exposure to both oxidizing agents, as well as an increase in carbonyls, derived products of protein oxidation six times higher in BLES treated with Fenton’s reaction compared to hypochlorous acid (*p* = 0.008), of which a significant increase in carbonyls from SP-B was shown, being 2.6 times higher by the Fenton’s reaction.

Therefore, the indirect role of meconium compounds on the inactivation of pulmonary surfactant is corroborated through the oxidation of both PL and surfactant proteins (SP-B and SP-C) mediated by elements resulting from the inflammatory process in the lung after aspiration.

## 6. Future Directions

The research tendencies regarding MAS have made it clear that it has areas of study that have not been explored. For example, there is still controversy about whether the increase in cytokines produced by meconium or those present in it may be associated with increased cell apoptosis [16] or even ponder the interaction of these cytokines with angiotensin, which could be considered as a direct biomarker of this injury [76]. Similarly, could the inhibition of alveolar macrophage phagocytosis and its respiratory burst response by meconium exposure influence the increase in cell damage? Because this action is known to help remove meconium microparticles and pathogens; however, decreased phagocytosis and its response could promote the apoptosis of these alveolar cells and increase the susceptibility to secondary lung infections in neonates [66].

The treatment of MAS is controversial; since its use does not clearly influence decreasing neonatal mortality incidence, monotherapies such as corticosteroids, antibiotics, and phosphodiesterase inhibitors have presented variable results over time in reducing clinical signs of MAS [7,17,114]. Thus, it might be necessary to explore the combined use or multimodal therapy of the different drugs, as has been observed with the combined use of surfactant with glucocorticoids or the use with antibiotics that can help reduce the inactivation of the surfactant by increasing resistance to it [109,115]. Likewise, explore the use of vasodilators not only to reduce the incidence of pulmonary hypertension but also to consider that they may avoid hypoxia and reduce acidosis in the newborn [116,117].

Finally, an area of opportunity that has not been fully explored is combining treatment with ventilatory assistance with other options. In this regard, it has been observed that the use of standard high-frequency jet ventilation or in combination with low-rate intermittent mandatory ventilation can decrease the level of bronchopneumonia and edema in the lung affected by the MAS in the canine model [118]. Moreover, the combination with surfactant can reduce the presence of lung lesions in the pig model [32,102,119]. It is suggested that using some central nervous system stimulants, such as caffeine, could be beneficial in increasing tidal volume in newborns with signs of hypoxia [120]. In the same way, explore the possibility of the combined use of the treatments previously described with the use of non-invasive ventilatory assistance that could promote recovery or even reduce lung injury. Data has yet to be presented to date, perhaps due to the lack of complete understanding of the pathophysiological mechanisms of MAS.

The contribution of experimental animal models of MAS in advancing knowledge of pathophysiology, the inflammatory process, the inactivation of surfactants, and the different therapeutic options are evident. It is important to note that many experimental studies on the pathophysiology of MAS have used adult laboratory animals without considering that MAS is a disease of the newborn and without considering possible differences in the inflammatory response between fetuses, neonates, and adult animals. In addition, to evaluate the importance of using heterologous instead of homologous meconium in experimental models. Therefore, neonatal animal models should be considered to evaluate the damage and inflammatory response, understand the pathogenesis and prevent MAS.

## 7. Conclusions

MAS, in veterinary medicine, is a pathological process affecting the newborn’s survival; therefore, early recognition could be a tool to reduce mortality in the newborn.

The main event in the MAS is the inflammatory process at the pulmonary level, which is related to the hyperreactivity of the alveolar epithelium due to the presence of meconium. Other meconium components also participate, such as proinflammatory cytokines that generate leukocyte chemotaxis and lead to epithelial destruction by inducing apoptosis. The inhibition of the phagocytic function of alveolar macrophages results from an oxidative process by reactive oxygen radicals. It is also associated with local immunosuppression, which may also be related to meconium components that inactivate and prevent the surface function of pulmonary surfactant and an immunological response by degrading its components.

Recognition of the pathophysiology of this process allows the possibility to design and plan strategies for its prevention and treatment to reduce the adverse effects on lung activity derived from the MAS and its influence on newborn survival.

## Data Availability

Not applicable.
